# Behçet-like Syndromes: A Comprehensive Review

**DOI:** 10.3390/dermatopathology13010007

**Published:** 2026-01-16

**Authors:** Gaia Mancuso, Igor Salvadè, Adam Ogna, Brenno Balestra, Helmut Beltraminelli

**Affiliations:** 1 Allergy and Clinical Immunology Service, Regional Hospital of Locarno, Ente Ospedaliero Cantonale (EOC), 6600 Locarno, Switzerland; 2 Department of General Internal Medicine, Regional Hospital of Locarno, Ente Ospedaliero Cantonale (EOC), 6600 Locarno, Switzerland; 3 Faculty of Biomedical Sciences, Università della Svizzera Italiana (USI), 6962 Lugano, Switzerland; 4 Department of General Internal Medicine, Regional Hospital of Mendrisio, Ente Ospedaliero Cantonale (EOC), 6850 Mendrisio, Switzerland; 5 Dermatology and Dermatopathology Unit, Ente Ospedaliero Cantonale (EOC), 6500 Bellinzona, Switzerland

**Keywords:** Behçet-like syndrome, Behçet’s disease, HLA-B51, autoinflammatory disorders, immune dysregulation, genetic predisposition, immunotherapy

## Abstract

Background: Behçet-like syndrome (BLS) refers to the presence of Behçet’s disease (BD) features occurring in association with distinct clinical–pathological conditions such as inborn errors of immunity, myeloproliferative disorders, infections, or drug exposure. BLS may differ clinically from BD and is increasingly recognized as a separate entity. Distinguishing BLS from primary BD is essential for appropriate management, and studying BLS may provide insights into BD pathogenesis. Objectives: To summarize clinical features, treatments, and genetic abnormalities reported in BLS, we reviewed all published cases up to January 2024. Methods: A systematic search of PubMed, Scopus, and Embase was performed using the terms “Behçet-like syndrome”, “Behçet-like disease”, and “Pseudo-Behçet disease”. We included English-language reports of patients > 12 years old with a defined underlying etiology and Behçet-like manifestations, defined by ≥2 ICBD criteria and/or gastrointestinal involvement, mucosal ulcers, thrombosis, or non-recurrent disease. Epidemiological, clinical, laboratory, histological, and treatment data were extracted and analyzed descriptively. Results: Of 679 publications, 53 met inclusion criteria, comprising 100 patients with BLS. The median age was 44 years (IQR 22–52), with a female predominance (1:2). Fifty-three percent were from non-European countries. A genetic disorder was identified in 70% of cases, while HLA-B51 was present in 10%. Frequent manifestations included skin lesions (68%), fever (56%), intestinal involvement (43%), and joint symptoms (43%). Treatments included glucocorticoids (65%), conventional DMARDs (32%), and biologics (22%), mainly anti-TNF agents. Antiviral/antibiotic therapy was used in 9% and chemotherapy in 15%. Two patients with trisomy-8 MDS underwent allogeneic stem cell transplantation. Conclusions: Diverse conditions—including monogenic diseases, immune defects, myeloproliferative disorders, infections, and drug-related reactions—can produce Behçet-like features. Our findings highlight differences in clinical expression and treatment response across BLS etiologies. Recognizing BLS is essential for appropriate management and may contribute to a deeper understanding of BD pathogenesis and future targeted therapies.

## Simple Summary

Behçet-like syndromes (BLSs) are conditions that present with symptoms similar to Behçet’s disease—such as oral and genital ulcers, fever, skin lesions, joint pain, and intestinal involvement—but arise in association with other underlying disorders. These include monogenic immune defects, myeloproliferative diseases, infections, or reactions to specific medications. Because BLS can resemble Behçet’s disease but requires different treatments, recognizing the underlying cause is essential for proper management. In this review, we summarize all published cases of BLS up to January 2024, highlighting their clinical features, genetic findings, and therapeutic approaches. Understanding BLS as a distinct group of conditions may also provide insights into the mechanisms that drive Behçet’s disease itself.

## 1. Introduction

Behçet-like syndrome (BLS) is defined as the presence of Behçet syndrome features, or Behçet disease (BD), occurring in association with a distinct clinical and/or pathological defined entity, such as various inborn errors of immunity (IEI), including autoinflammatory conditions related to dysregulation of the nuclear factor kappa B (NF-κB) pathway (e.g., TNFAIP3, NFKB1, RELA, IKK gamma mutations) or DADA2, CSF2, and LIG4 mutations [1]. BLS has also been largely described in association with myeloproliferative and myelodysplastic disorders [1,2]. Moreover, some microbial infections, such as Mycobacterium Tuberculosis (MT) and viral infections, can trigger the onset of BLS in genetically susceptible subjects [3]. The development of a BLS has even been reported following drug administration [4].

The clinical symptoms in BLS—such as oral and genital aphthous ulcers, fever, skin lesions, arthralgia/arthritis, ocular inflammation, gastrointestinal ulceration, and vascular involvement—may occur before the onset of the associated diseases [5,6,7] or later on in the disease course [8,9], further expanding the phenotypic spectrum of the associated entities.

BLS does not exhibit specific or pathognomonic histopathological features. As in classical BD, biopsy findings are often nonspecific and may include neutrophilic infiltrates, lymphocytic vasculitis, or ulceration, but none of these patterns reliably distinguish BLS from BD or from other inflammatory or ulcerative conditions. For this reason, the diagnosis of BLS cannot rely on histopathology alone. Instead, it requires the coexistence of Behçet-like clinical manifestations together with a clearly identifiable underlying condition—such as a monogenic autoinflammatory disorder, myelodysplastic syndrome, infection, or drug-related immune dysregulation. Histopathology may support the exclusion of mimickers (e.g., pyoderma gangrenosum, Lipschütz ulcer), but it does not define BLS.

The treatment strategy of BLS differs based on the underlying aetiology: NSAIDs or traditional immunosuppressive therapies frequently fail to relieve the symptoms of BLS [10,11,12,13,14,15,16,17]. Thus, it is essential to distinguish BLS from primary BD in order to ensure appropriate treatment.

This review highlights the clinical characteristics, genetic alterations, and therapeutic approaches of all clinical cases of BLS and associated conditions published up to January 2024, aiming to better define the key features of BLS, particularly those linked to monogenic diseases. Such extensive analysis could enhance our understanding of the pathogenesis of BD and pave the way for further research into its pathophysiology and potential therapeutic targets.

## 2. Methods

We conducted a literature review of all relevant reports on patients affected by Behçet-like syndrome (BLS) and associated diseases by searching the PubMed, Scopus, and Embase databases in January 2024. The search terms included “Behçet-like syndrome”, “Behçet-like disease”, and “Pseudo-Behçet disease”, with a language restriction to English. We retrieved and reviewed all potentially relevant abstracts and examined the references of the obtained publications to identify additional reports. Non-peer-reviewed reports were excluded. We included only patients with a clearly defined etiological entity and Behçet-like disease, which was defined by the presence of at least two of the International Criteria for Behçet’s Disease (ICBD) [9] and/or a combination of gastrointestinal involvement, oral and/or genital aphthous ulcers, thrombosis, and/or the absence of demonstrated recurrence over time.

We excluded: (1) patients fulfilling full ISG or ICBD criteria for Behçet’s disease (BD) [9]; (2) pediatric cases < 12 years; (3) reports lacking a clearly defined underlying etiology; (4) non-English publications; (5) conference abstracts without sufficient clinical detail. We collected data on sex, age at diagnosis, underlying genetic disorders, clinical manifestations, diagnostic procedures, and treatments. Specifically, we gathered information on the following clinical features: non-infectious fever, skin abnormalities, arthritis, ocular manifestations, gastrointestinal symptoms, peripheral or central neurological involvement, venous or arterial thrombosis, associated diseases, diagnostic procedures, and treatments. Laboratory data were included, when available, encompassing blood counts, C-reactive protein (CRP), erythrocyte sedimentation rate (ESR), serum creatine kinase levels, hepatitis C virus (HCV) and HIV serologies, immunological markers (anti-neutrophil cytoplasmic and antinuclear antibodies, cryoglobulinemia), and human leukocyte antigen B51 (HLA-B51) status. We also reviewed endoscopic abnormalities and histopathological findings. Because the evidence consisted exclusively of case reports and small case series, we assessed risk of bias using adapted CARE and JBI criteria, focusing on clarity of case definition, completeness of clinical description, diagnostic certainty, and reporting of outcomes. Given the descriptive nature of the data, no cases were excluded based on quality, but reporting limitations were considered when interpreting results.

Descriptive statistics were employed to ascertain the frequencies and central tendencies of the cohort. Data are expressed as median (interquartile range [IQR]: 25° and 75° percentiles) for quantitative variables and number (percentage) for categorical variables. To compare continuous variables across etiologic groups, we adopted the Kruskal–Wallis test, while for the categorical variables we used the Chi-square test.

## 3. Results

The database search identified 679 publications, of which 53 met the inclusion and exclusion criteria, reporting a total of 100 patients with Behçet-like syndrome (Table 1).

### Characteristics of Patients with Behçet-like Syndrome

The 100 patients included in this review illustrate that BLS represent a heterogeneous group of disorders sharing a Behçet-like phenotype but differing substantially in their underlying mechanisms and clinical expression. The median age at diagnosis was 44 years (IQR 22–52), with symptoms often beginning much earlier (median onset 25 years). Women were more frequently affected (61%), and over half of the cohort originated from non-European countries, particularly Turkey and Japan. Despite this geographic distribution, no ethnicity-specific clinical patterns emerged, and NF-κB–related mutations (A20, NEMO, RELA) appeared evenly distributed across populations.

A defined genetic abnormality was identified in 70% of cases, most commonly involving the NF-κB pathway (TNFAIP3, NFKB1, RELA, IKBKG), but also ADA2, CSF2, and LIG4. HLA-B51 positivity was uncommon (10%), supporting the notion that BLS differs immunogenetically from classical Behçet disease.

Fever was the most frequent systemic manifestation (56%), but its distribution varied markedly by etiology (Table 2 and Table 3). Fever was notably less frequent in the NEMO group and had a higher incidence in infectious diseases when compared to other etiologies.

Mucocutaneous involvement was nearly universal, but its expression varied:

Oral ulcers (including aphthous lesions) occurred in 88% of patients and were particularly prominent in monogenic NF-κB–related disorders (A20, NEMO, RELA).

Genital ulcers were less frequent (57%) but tended to cluster in NEMO-related and infection-associated BLS.

Skin lesions (68%) were dominated by pseudofolliculitis-like eruptions and erythema nodosum. Erythema nodosum was more common in infection-related BLS, reflecting a reactive inflammatory pattern. No cases of urticaria were reported, helping differentiate BLS from other autoinflammatory or allergic conditions. Pathergy was uncommon (11%) and did not distinguish etiologic groups. Joint involvement (43%) showed one of the clearest etiologic patterns:

A20 haploinsufficiency was strongly associated with arthromyalgia and polyarthritis (*p* = 0.030 and *p* = 0.010), suggesting a more systemic autoinflammatory phenotype.

NEMO-related BLS showed minimal articular involvement, reinforcing its mucocutaneous predominance. Gastrointestinal involvement was common across the cohort but showed a striking concentration in patients with hematologic disorders:

Inflammatory bowel disease–like features were present in 43% of all patients.

Trisomy 8–associated myelodysplastic syndromes showed the highest rates of intestinal ulceration, bleeding, and stenosis/obstruction.

A20-related disease also showed a tendency toward intestinal ulcers, though less severe than in hematologic BLS.

Ocular involvement (19%) was disproportionately represented in infection-related BLS, particularly tuberculosis-associated cases.

Neurologic manifestations (14%) were scattered across etiologies without a clear pattern.

Thrombosis occurred in 16% of patients, but reports rarely specified whether histopathology resembled classical Behçet vasculitis, limiting interpretation.

A minority of patients (6%) exhibited benign inflammatory lymphoproliferation mimicking PFAPA (Periodic Fever, Aphthous Stomatitis, Pharyngitis, Adenopathy), highlighting the overlap between autoinflammatory syndromes.

Table 2 and Table 3 highlight the distribution of symptoms and signs across the etiologic groups.

A total of 83% of patients received treatment. Glucocorticoids were administered to 65% of patients, and the conventional disease-modifying antirheumatic drugs (cDMARDs) were used in 32%, including colchicine, 5-aminosalicylic acid (5-ASA)/mesalazine, mycophenolate mofetil, dapsone, apremilast, and methotrexate. Some patients (9%) received antiviral and antibiotic therapy, whilst chemotherapy (azacitidine, venetoclax) was necessary in 15% of cases. The biologic disease-modifying antirheumatic drugs (bDMARDs) were used in 22% of patients, primarily anti-TNF-α (16 cases), followed by anti-IL1 (3 cases), anti-IL17 (2 cases), anti-IL23 (1 case), and anti-CD20 (1 case). Allogeneic hematopoietic stem cell transplantation was required in two cases due to trisomy 8 myelodysplastic syndrome.

## 4. Discussion

This review demonstrates that BLS encompasses a heterogeneous group of conditions that mimic BD but arise from distinct pathogenic mechanisms. Recognizing these patterns is essential because management differs substantially from classical BD.

Despite our systematic review of the literature and the broad heterogeneity of the reported clinical presentations, we were unable to propose a unified or universally applicable definition of Behçet-like syndrome (BLS). The conditions described share clinical features reminiscent of Behçet disease but do not fulfill PEDBD, ISG, or ICBD diagnostic criteria and occur in association with a wide range of underlying disorders. This variability currently prevents the formulation of formal diagnostic criteria or a standardized definition.

**Figure 1:**
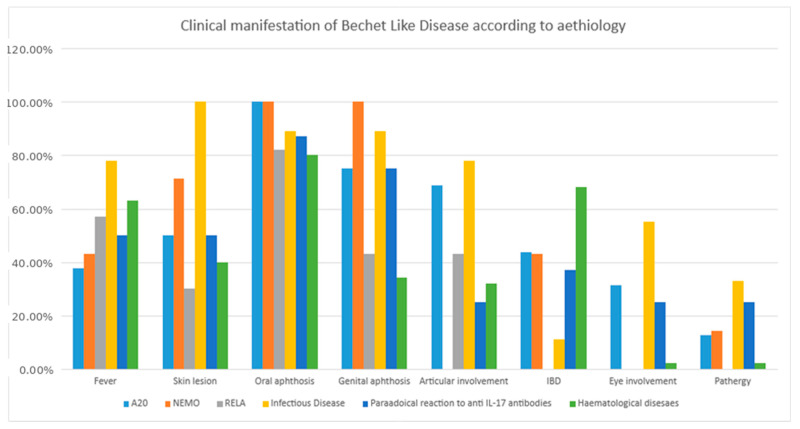
Clinical characteristics of patients with Behçet-like syndrome according to aetiology.

We support the current conceptualization of BLS as a clinical condition in which features typical or compatible with Behçet disease—such as recurrent oral or genital aphthosis, cutaneous lesions, arthritis, uveitis, vasculitis, or intestinal ulceration—occur in patients who do not fulfill established diagnostic criteria for BD and who present a clearly identifiable underlying etiology [9,56]. These etiologies include monogenic autoinflammatory disorders, inborn errors of immunity, myelodysplastic or myeloproliferative diseases, infections, or paradoxical reactions to biologic therapies [1,2,3,4,5,6]. The present literature review allows us to describe different subgroups of BLS, associated with different underlying aetiological mechanisms. Interestingly, each subgroup seems to have some distinctive clinical features (Figure 1). We discuss below the features of 4 different groups of BLS. In this framework, BLS represents a secondary or syndromic entity, heterogeneous in its causes and pathogenic mechanisms, yet unified by a clinical phenotype that resembles BD without constituting a primary form of the disease.

### 4.1. Genetically Related Behçet-like Syndrome

**Figure 2:**
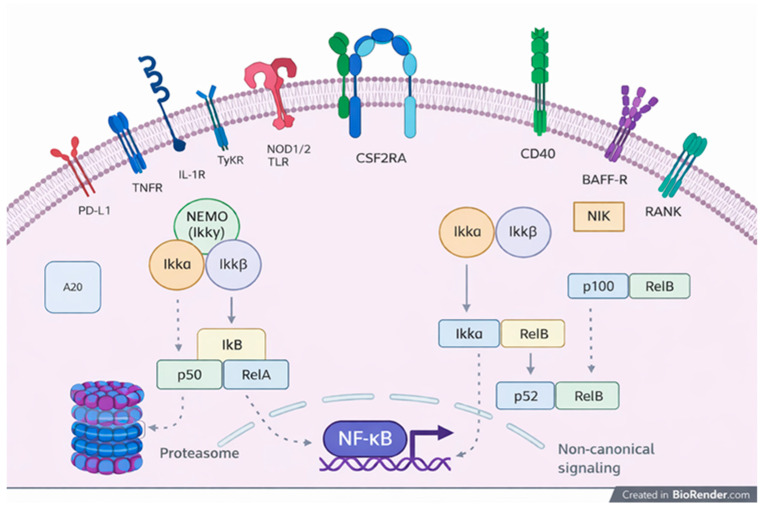
NF-kB signaling. NF-kB is activated, other than by antigen recognition, by TLR ligands and by many cytokines belonging to the TNFR and IL 1R families through two pathways: the canonical and the non-canonical pathways.

Genetic mutations play a significant role in the pathogenesis of BD, and several monogenic conditions mimic its clinical manifestations. The NF-κB signaling pathway is central to immune cell activation and inflammation (Figure 2). Genetic mutations affecting the NF-κB pathway, notably in genes like A20, NEMO, and RELA, are key contributors to BLS. Mutations in A20, a protein essential for suppressing NF-κB activity, lead to an imbalance in immune responses, causing spontaneous inflammation and an increased response to TNF-α, IL-1β, and IL-18, exacerbating inflammatory processes in BLS patients [12,13].

Monogenic disorders affecting the NF-κB pathway (A20, NEMO, RELA) can closely mimic BD. These mutations disrupt immune regulation and promote exaggerated responses to TNF-α, IL-1β, and IL-18 [12,13,14,15,16], resulting in a Behçet-like inflammatory phenotype. In our synthesis, A20 haploinsufficiency emerged as the most clinically expressive condition, with a combination of recurrent oral ulcers, genital aphthosis, polyarthritis, and gastrointestinal ulceration. The prominence of articular involvement in A20-related disease suggests a systemic autoinflammatory signature that differs from classical BD.

NEMO-related disease, in contrast, tended to present with mucocutaneous features and relatively limited systemic inflammation. RELA haploinsufficiency [13,15,18,19,21,52,57,58,59,60,61,62,63] showed an intermediate phenotype, with mucosal ulceration as the dominant feature. These observations reinforce the need to consider monogenic causes in early-onset, familial, or treatment-refractory Behçet-like presentations, as the therapeutic approach may differ substantially from BD-oriented immunosuppression.

Similarly, mutations in the ADA2 gene, underlying DADA2 disease, could also lead to recurrent oral/genital ulcers and other BD-like symptoms, complicating the diagnostic process and underscoring the need for genetic testing in atypical cases [20,64,65].

For monogenic BD-like diseases, particularly those involving NF-κB pathway mutations, therapies targeting inflammatory cytokines such as TNF-α or IL-1 may be beneficial. In cases where genetic mutations like A20, NEMO, DADA2 or RELA are identified, tailored therapies that modulate the immune response to prevent excessive inflammation should be prioritized [66].

### 4.2. Infection-Related Behçet-like Syndrome

Infections caused by viruses and mycobacteria can act as triggers for Behçet-like manifestations, particularly in genetically predisposed individuals. This observation supports the molecular mimicry hypothesis, whereby pathogens such as Mycobacterium tuberculosis (MT) and herpes simplex virus (HSV) share antigenic homology with host proteins, including heat shock protein 60 (HSP60), which is overexpressed in active BD lesions and may function as an autoantigen [3,6,67]. In this context, infectious agents may amplify innate immune activation and precipitate a Behçet-like phenotype, especially in individuals carrying susceptibility factors such as HLA-B51.

MT-associated BLS represents the best-documented infectious subgroup. These cases predominantly affect women, with a median onset age of approximately 45 years, and typically present with bipolar aphthosis, erythema nodosum, joint involvement, and ocular inflammation. The temporal relationship between tuberculosis and BLS varies considerably: symptoms may arise concurrently with active infection, years after its onset, or—less commonly—precede the diagnosis of MT infection. This variability highlights the need to consider occult or past infections when evaluating Behçet-like presentations.

Several viral pathogens—including HIV, EBV, HSV, and SARS-CoV-2—have also been implicated in BLS [31,32,33,34,68]. Compared with MT-related cases, viral BLS tends to have a shorter latency between infection and symptom onset. Fever, oral aphthosis, and pseudofolliculitis-like lesions were consistently reported across viral cases, while ocular inflammation was frequent, but major organ involvement was generally absent. The predominance of mucocutaneous and systemic inflammatory features suggests that viral infections may act as potent but transient immune triggers.

From a clinical perspective, these findings underscore the importance of identifying infectious etiologies before initiating immunosuppression. In most cases, targeted antimicrobial therapy leads to improvement of Behçet-like symptoms, confirming the reactive nature of the inflammatory process. However, persistent inflammation after pathogen eradication may require adjunctive immunomodulation, and TNF inhibitors have been used successfully in selected cases [47,68]. Recognizing infection-related BLS is therefore essential to avoid misdiagnosis, prevent inappropriate treatment, and ensure timely management of the underlying infection.

### 4.3. Myeloproliferative Disorders-Related Behçet-like Syndrome

Proinflammatory cytokines, including IL-1β, TNF-α, and IL-18, play a pivotal role in the pathogenesis of BD [2,5,44,46,69]. In myeloproliferative neoplasms (MPN), which are frequently associated with BD-like symptoms, there is a significant increase in inflammatory cytokine production [70,71], contributing to the chronic inflammatory state observed in affected patients. The interaction between myelodysplastic cells and T-cells may also play a key role in the development of autoimmune features in MPN-associated BLS. Forty-four cases of hematologic neoplasms with BLS have been reported, predominantly in Japanese patients (median age: 68 years). Most cases exhibited an incomplete BD phenotype [42,43,49,72,73,74,75], particularly the intestinal variant, frequently associated with trisomy 8 in myelodysplastic syndrome (MDS). Intestinal involvement (68%) was complicated by obstructions or stenosis in 18% of cases [45,50].

Chemotherapeutic treatment and immunosuppressive therapy, including prednisolone, cyclosporine A, TNF-α inhibitors, and mesalazine, provided symptom relief but were often insufficient for complete disease control. Allogeneic hematopoietic stem cell transplantation (allo-HSCT) successfully treated both MDS and BD-like symptoms in two cases, suggesting its potential as a curative option. Moreover, the literature data report that immunosuppressive therapies such as ruxolitinib may alleviate BLS symptoms in MPN patients [7,51,76,77,78,79,80,81,82]. Overall, these findings emphasize that gastrointestinal Behçet-like disease in older adults—particularly when accompanied by cytopenias or systemic inflammation—should prompt evaluation for underlying MDS or MPN. Early recognition is essential, as management differs substantially from classical BD and may require hematologic intervention.

### 4.4. BLS, Paradoxical Reactions, and BD Exacerbations Induced by Biological Therapies

Cytokine imbalance may also underlie paradoxical Behçet-like reactions induced by biologic therapies [3,4,48,83,84]. De novo BD or exacerbations of pre-existing BD have been reported following treatment with several biologic agents, particularly anti-IL-17 therapies such as secukinumab and ixekizumab [22,23,24,25,29,85,86,87,88,89,90]. Similar Behçet-like presentations have been described as immune-related adverse events (irAEs) associated with immune checkpoint inhibitors [3,30,91], reflecting the profound immune modulation induced by these treatments.

Clinical manifestations in paradoxical BLS are heterogeneous but frequently include fever and skin lesions (50%), as well as oral and genital ulcers (87% and 75%, respectively). Anterior uveitis and gastrointestinal involvement were reported in 25% and 37.5% of cases, respectively, indicating that paradoxical reactions may reproduce the full spectrum of BD-like inflammation.

These observations have important clinical implications. Patients receiving biologic therapies should be monitored closely for new mucocutaneous or ocular symptoms, as early recognition and timely discontinuation of the offending agent can prevent progression and reduce morbidity [4,22,23,25,29]. Corticosteroids remain the mainstay of treatment and lead to symptom resolution in most cases [12,13,14,15,25]. In selected situations, switching to an alternative biologic class may be necessary to maintain control of the underlying disease while avoiding recurrence of Behçet-like manifestations.

### 4.5. Strengths and Limitations

This review is, to our knowledge, the first to comprehensively synthesize all published cases of BLS up to January 2024. Despite the heterogeneity of the included reports, the aggregated data offer valuable insights into the mechanisms that generate Behçet-like phenotypes and help delineate clinically relevant subgroups.

However, the evidence is inherently limited. All data derive from case reports and small case series, which are subject to publication bias, incomplete reporting, and variability in diagnostic work-up. Clinical features were not uniformly described, and histopathologic confirmation was often lacking. As a result, frequency estimates and inter-group comparisons should be interpreted cautiously, and causal relationships cannot be inferred.

**Figure 3:**
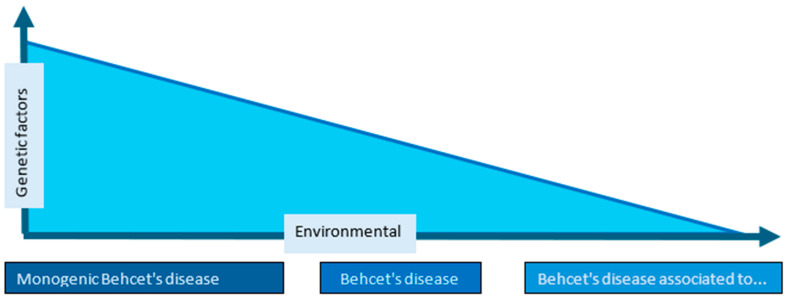
Simplified etiopathogenetic hypothesis of Behcet’s disease spectrum.

The overlap between BD and BLS also raises conceptual questions. The low prevalence of HLA-B51 positivity and the incomplete penetrance observed in familial BD suggest that genetic background alone does not determine phenotype [17,18,19,20,21,52,56,57,58,59,60,61,62,63,64,65,92]. Environmental triggers, infections, hematologic disorders, and cytokine dysregulation likely modulate disease expression, supporting the idea of a spectrum rather than a strict dichotomy (Figure 3).

### 4.6. Practical Recommendations for Clinicians

Based on the patterns identified in this review, several practical considerations emerge:

Suspect BLS rather than BD when Behçet-like features occur with cytopenias, recurrent infections, early-onset autoinflammation, or recent exposure to biologics.

Investigate monogenic autoinflammatory diseases (A20, RELA, DADA2, NEMO) in early-onset or familial cases with prominent mucocutaneous involvement.

Evaluate for myelodysplastic syndromes, particularly trisomy 8, in patients with intestinal ulcers, unexplained cytopenias, or poor response to BD-oriented therapy.

Consider infectious triggers—especially tuberculosis and chronic viral infections—when fever and erythema nodosum predominate.

Tailor treatment to the underlying etiology, as management differs substantially from classical BD.

Monitor for paradoxical reactions in patients receiving IL-17 inhibitors or immune checkpoint inhibitors.

Recognizing BLS as a distinct clinical construct is essential to avoid misdiagnosis, prevent inappropriate immunosuppression, and guide targeted therapy. Moreover, the study of BLS may provide valuable insights into BD pathogenesis and help identify new therapeutic targets.

## 5. Conclusions

This extensive literature review highlights the characteristics of the different entities included in BLS, related to classical and recently described autoinflammatory syndromes, myeloproliferative diseases, viral and mycobacterial infections, as well as paradoxical reactions secondary to drug treatment. The typical clinical manifestations of BD help us to conceptualize that BD is the expression of a complex cytokine balance disorder, in the context of a genetic predisposition, sometimes influenced by environmental factors, and with possible interactions with other inflammatory pathways.

Given the potential severity of BLS, being aware of the different BLS entities described in this article is fundamental for the correct management of patients. Furthermore, the development of effective therapeutic strategies remains an urgent priority. Future research focusing on the molecular mechanisms underlying immune dysregulation in BLS is essential to identify optimal targeted treatments and improve patient outcomes.

## Figures and Tables

**Table 1:** Literature data referring to Behçet-like Syndrome patients.

No	References	Nationality/Ethnicity	Study Population	Genetic Hallmark	HLA B51 Prevalence	Clinical Associations Discussed
1	[13]	Irish	5 pts	RELA haploinsufficiency	nr	RELA Truncating Mutation Mucocutaneous Ulcerative Condition
2	[15]	Canadian	15 pts	RELA haploinsufficiency	nr	RELA haploinsufficiency and mucocutaneous ulceration and autoinflammation
3	[18]	na	10 pts	NEMO mutation	na	NeMO mutations: a rare cause of monogenic Behçet-like disease
4	[14]	Chinese	2 pts	A20 Haploinsufficiency	nr	A20 Haploinsufficiency and Intestinal Behcet’s Disease-Like Symptoms
5	[19]	Danese	1 pt	ERAP1 mutation	no	Oral involvement, but also involvement of the lower part of the GI tract, including duodenum and colon, where biopsies showed granuloma
6	[11]	Chinese	1 pt	A20 Haploinsufficiency	nr	TNFAIP3 mutation and autoimmune syndrome resembling Behçet’s disease
8	[20]	Turkish	2 pts	ADA2	no	Phenotypic variability, including Behçet’s disease-like manifestations, in DADA2 patients due to a homozygous c.973-2A > G splice-site mutation
9	[21]	Dutch	1 pt	CSF2 gene (c.130A > C, p.N44H	yes	GM-CSF gain-of-function mutation in a family suffering from a Behçet’s disease-like disorder
10	[8]	Turkish	1 pt	homozygous p.Arg871His (c.2612G > A) mutation in LIG4.	yes	LIG4 Mutation in a Patient with a Phenotype Mimicking Behçet’s Disease
11	[22]	NR	1 pt	NR	yes	Behçet’s-like disease during secukinumab treatment: new paradoxical reaction
12	[23]	NR	1 pt	NR	yes	Pyoderma gangrenosum and Behçet’s-like disease induced by secukinumab: a paradoxical drug reaction
13	[24]	Nr	1 pt	Nr	Yes	Two cases of Behçet’s disease developed during treatment with secukinumab
14	[24]	Nr	1 pt	Nr	No	Two cases of Behçet’s disease developed during treatment with secukinumab
15	[25]	Nr	1 pt	Nr	No	Interleukin-17A inhibitor-induced Crohn’s disease/Behçet’s disease-like lesions
16	[12]	Caucasica e Turca	11 pts	A20 Haploinsufficiency	yes (in some patients)	Loss-of-function mutations in TNFAIP3 leading to A20 haploinsufficiency cause an early-onset autoinflammatory disease
17	[26]	nr	1 pts	*NEMO*	yes	Incontinentia pigmenti and Behçet’s disease
18	[27]	nr	1 pts	*NEMO*	no	Incontinentia pigmenti and bipolar aphthosis: an unusual combination
19	[8]	nr	1 pts	LIG4	yes	A Novel Missense LIG4 Mutation in a Patient with a Phenotype Mimicking Behçet’s Disease
20	[28]	African	1 pt	nr	no	Behcet’s disease in a patient with immunodeficiency virus infection.
21	[29]	nr	1 pt	nr	no	Behcet’s-like disease in a patient treated with Ixekizumab for chronic plaque psoriasis
22	[4]	Nr	1 pts	nr	yes	Secukinumab-induced syndrome: a report of two cases
23	[4]	Nr	1 pts	nr	no	Secukinumab-induced syndrome: a report of two cases
24	[30]	nr	1 pt	nr	ne	Behcet’s-like syndrome following pembrolizumab
25	[31]	Japanese	1 pt	nr	no	Tuberculous Lymphadenitis and the Appearance of Behçet’s Disease-like Symptoms
26	[32]	nr	1 pt	nr	no	Pseudo-Behcet’s disease associated with tuberculosis
27	[33]	nr	1 pt	nr	nr	Poncet’s disease presenting as pseudo-Behçet’s disease
27	[34]	Chinese	1 pt	nr	no	Behçet’s disease-like syndrome secondary to TB infection
27	[35]	nr	1 pts	nr	nr	Pemphigus vulgaris
28	[36]	nr	1 pts	nr	nr	Behcet’s-like syndrome, idiopathic CD4+ T-lymphocytopenia
29	[37]	Korean	1 pts	nr	no	Chronic Active Epstein–Barr Virus Infection-
30	[38]	nr	1	nr	nr	Behçet’s disease-like clinical manifestations of chronic lymphocytic leukemia during good response to ibrutinib
31	[2]	Japanese	1 pts	nr	no	Behçet’s disease-like illness associated with acute monocytic leukemia
32	[39]	nr	1	47, XY, +8 karyotype	no	Behçet’s Disease-like Symptoms Associated with Myelodysplastic Syndrome with Trisomy 8: A Case Report and Review of the Literature
33	[40]	Caucasian	1 pt	nr	no	Behçet’s-like adverse event or inaugural Behçet’s disease after SARS-CoV-2 mRNA-1273 vaccination?
34	[41]	nr	1 pt	47, XX, +8	no	Intestinal Behçet’s-like disease accompanied with myelodysplastic syndrome involving trisomy 8
35	[7]	nr	1 pt	47, XX, +8, −20, −21,	no	Myelodysplastic syndrome associated with intestinal Behçet’s-like disease
36	[42]	Hispanic	1 pt	loss of chromosomes 3, 5, 9, 12 and 20	no	Myelodysplastic syndrome presenting as a Behçet’s-like disease with aortitis
37	[43]	nr	1 pt	trisomy 8 and 9	no	Trisomy 8-positive Polycythemia Vera Complicated with Intestinal Behçet’s-like Disease
40	[44]	Japanese	1 pt	karyotic analysis showed 47, XX, der (1:18)(q10;q10), inv (9) (p12q13) (14/20 cells), 46, XX, inv (9) (p12q13) (6/20 cells)	no	Trisomy 8 associated with Behçet’s-like disease in myelodysplastic syndrome
41	[45]	nr	1 pt	47, XY, +8 in 1 out of 20 cells	no	Refractory Intestinal Behçet-Like Disease Associated with Trisomy 8 Myelodysplastic Syndrome
42	[46]	Japanese	1 pt	complex karyotype abnormality including trisomy 8	no	A Case of Myelodysplastic Syndrome with Intestinal Behçet’s Disease-Like Symptoms
43	[47]	Japanese	1 pt	nr	yes	Single episode of Behcet’s disease-like symptoms caused by herpes simplex virus reactivation
44	[5]	Japanese	1 pt	E148Q variant of MEFV gene and trisomy 8	nr	A case of Behçet’s-like disease associated with trisomy 8–positive myelodysplastic syndrome carrying MEFV E148Q variant presented with periodic fever
45	[17]	Japanese	4 pts	HA20 Haploinsufficiency	nr	Haploinsufficiency of A20 caused by a novel nonsense variant or entire deletion of TNFAIP3 is clinically distinct from Behçet’s disease
46	[48]	nr	1 pt	46XX	no	Behçet’s-like syndrome associated with aplastic anemia
47	[49]	nr	1 pt	monosomy 7	nr	Intestinal Behçet’s disease-like ulcers associated with myelodysplastic syndrome with monosomy 7
48	[50]	na	11 pts	trisomy 8	na	Behcet’s-like disease in myelodysplastic syndrome
49	[51]	na	11 pts	trisomy 8 (13 pts)	na	Behcet disease-like syndrome and Myelodysplastic syndrome
50	[52]	nr	2 pts	NEMO mutation	no	NEMO mutation as a cause of familial occurrence of Behçet’s disease in female patients
51	[53]	nr	2 pts	NEMO mutation	nr	Flare-up of incontinentia pigmenti in association with Behçet’s disease
52	[54]	nr	3 pts	NEMO mutation	nr	Concurrence of Incontinentia Pigmenti and Behçet’s Disease.
53	[55]	nr	1 pt	nr	nr	Multiple cutaneous and mucocutaneous lesions as manifestations of pseudo-Behçet’s disease

Abbreviations: nr: not reported; na: not applicable.

**Table 2:** Clinical characteristics of patients (n = 100) with Behçet-like syndrome.

Age of Onset	25 (IQR 11–48)
Gender	61% female
Ethnicity	53% non-European
Underlying genetic disorder	70%
HLA-B51 Haplotype	10%
Clinical manifestations	%
Fever	56
Skin lesions	68
Articular involvement	43
Oral aphthosis	88
Genital aphthosis	57
Pathergy	11
Thrombosis	16
Ocular lesion	19
Central Nervous System	14
Intestinal Bowel Disease	43
Treatment	%
Glucocorticoids	65
cDMARDs	32
bDMARDs	22
AlloHTC	2
Etiology	%
Monogenic disease	32
Hematological disease	45
Paradoxical reaction	9
Infectious disease	8
Miscellaneous	6

**Table 3:** Clinical manifestations in BLS in blue are clinical manifestations according to the international criteria for Behçet disease scoring system [9] (* *p* < 0.05).

	A20	NEMO	RELA	Hematologic Neoplastic Diseases	Infections	Paradoxical Reactions
Ocular lesions	31%	0%	0%	2%	55%	25%
Genital aphthosis	75%	100%	43%	34%	89%	75%
Oral aphthosis	100%	100%	82%	80%	89%	87%
Skin lesions	50%	71%	30%	40%	100%	50%
Neurological manifestations	19%	0%	14%	5%	11%	0%
Vascular manifestations	12.5%	0%	0%	13%	0%	12%
Positive pathergy test	12.5%	14%	0%	2%	33%	25%
Intestinal Bowel Disease	43.75%	43%	0%	68%	11%	37%
Articular lesion	68.50% *	0%	43%	32%	78%	25%
Fever	37.50%	43%	57%	63%	78%	50%

## Data Availability

The data that support the findings of this study are available from the corresponding author upon reasonable request.
